# Bipolar disorder comorbid with alcohol use disorder: focus on neurocognitive correlates

**DOI:** 10.3389/fphys.2015.00108

**Published:** 2015-04-07

**Authors:** Vicent Balanzá-Martínez, Benedicto Crespo-Facorro, Ana González-Pinto, Eduard Vieta

**Affiliations:** ^1^Teaching Unit of Psychiatry, Deparment of Medicine, School of Medicine, La Fe University and Polytechnic Hospital, University of Valencia, CIBERSAM, ISNPRValencia, Spain; ^2^Department of Psychiatry, School of Medicine, University Hospital Marqués de Valdecilla, University of Cantabria-IDIVAL, CIBERSAMSantander, Spain; ^3^Álava University Hospital, CIBERSAM, University of the Basque CountryKronikgune, Vitoria, Spain; ^4^Barcelona Bipolar Disorders Program, Institute of Neurosciences, University of Barcelona, IDIBAPS, CIBERSAMBarcelona, Spain

**Keywords:** bipolar disorder, comorbidity, addiction, alcohol use disorders, neurocognition, staging, systems biology

## Abstract

Bipolar disorder (BD) and alcohol use disorders (AUDs) are usually comorbid, and both have been associated with significant neurocognitive impairment. Patients with the BD-AUD comorbidity (dual diagnosis) may have more severe neurocognitive deficits than those with a single diagnosis, but there is paucity of research in this area. To explore this hypothesis more thoroughly, we carried out a systematic literature review through January 2015. Eight studies have examined the effect of AUDs on the neurocognitive functioning of BD patients. Most studies found that BD patients with current or past history of comorbid AUDs show more severe impairments, especially in verbal memory and executive cognition, than their non-dual counterparts. Greater neurocognitive dysfunction is another facet of this severe comorbid presentation. Implications for clinical practice and research are discussed. Specifically, the application of holistic approaches, such as clinical staging and systems biology, may open new avenues of discoveries related to the BD-AUD comorbidity.

## Neurocognitive dysfunction is a core feature of bipolar disorder

Bipolar disorder (BD) is associated with significant morbidity, premature mortality and functional disability (Salomon et al., 2013; Conus et al., 2014). The major sources of this disability seem to be episode density, psychotic features, subclinical depression, sustained neurocognitive deficits, comorbidities, medication side effects, low premorbid functioning and weak social support (Sanchez-Moreno et al., 2009a).

It is well established that BD is associated with neurocognitive deficits that persist into euthymia after episode resolution, and thus represent a core symptom of the illness (Balanzá-Martínez and Dias, 2013). Several meta-analysis have revealed impairments in the broad domains of attention, processing speed, verbal memory and executive functions, with relative preservation of verbal abilities and intelligence (Torres et al., 2007; Bora et al., 2009; Bourne et al., 2013). This pattern of deficits is similar to that in schizophrenia (SZ), although less severe in magnitude (Daban et al., 2006; Sánchez-Morla et al., 2009). The variability in the degree and pattern of cognitive functioning among BD patients is also more pronounced than in SZ, and it has been estimated that 30–60% of euthymic BD patients show clinically relevant deficits (Martino et al., 2008). Moreover, in population-based studies, BD has been associated with increased risk for later development of dementia, especially in middle-age adults (Wu et al., 2013). This risk seems to increase with the number of episodes (Kessing and Andersen, 2004) and is independent of confounding variables such as comorbidities (Wu et al., 2013).

There is also growing evidence that neurocognitive impairments are major predictors of BD patients' long-term functional outcomes (Tabarés-Seisdedos et al., 2008; Wingo et al., 2009). Therefore, neurocognitive improvement represents a therapeutic target in BD (Fuentes-Durá et al., 2012). There is pressing need to develop interventions specifically addressed to ameliorate these deficits by means of pro-cognitive medications (Dias et al., 2012) and cognitive training and rehabilitative strategies, such as functional remediation (Torrent et al., 2013).

Persistent neurocognitive deficits (Balanzá-Martínez et al., 2005) likely result from the combination of genetic and environmental risk factors, as well as neurodevelopmental and neuroprogressive processes (Goodwin et al., 2008). Neurocognitive impairment may increase with illness progression (Robinson and Ferrier, 2006; Bourne et al., 2013) and history of psychotic symptoms (Selva et al., 2007; Martínez-Arán et al., 2008; Brissos et al., 2011), but it is also found in healthy first-degree relatives of patients with BD, although at a lesser degree (Arts et al., 2008; Balanzá-Martínez et al., 2008). Subsyndromal depressive symptoms, comorbidites and side effects of medications may compound and further worsen these deficits yet cannot fully explain them (Balanzá-Martínez et al., 2010).

The relative contribution of psychiatric comorbidities to BD patients' neurocognition has received limited attention. Dual diagnosis is the concomitant or comorbid presentation of a substance use disorder (SUD) or an alcohol use disorder (AUD) and another psychiatric condition. Patients with dual diagnosis represent a clinical population of special interest because BD is highly comorbid with addictions (Cerullo and Strakowski, 2007; Schoepf and Heun, 2014) and prolonged heavy use of alcohol and other substances is associated with persistent neurocognitive and brain abnormalities (Cunningham and McCambridge, 2012). Clearly, this relevant issue requires further examination.

## The bipolar—alcohol comorbidity

Several epidemiological and clinical studies have consistently found high rates of comorbid AUD (i.e., alcohol abuse or dependence) among BD patients (Merikangas et al., 2007; Mitchell et al., 2007; Oquendo et al., 2010). Indeed, BD is the DSM Axis I disorder most strongly associated with AUDs (Regier et al., 1990; Kessler et al., 1997). In a recent meta-analysis, lifetime prevalence of AUDs affected approximately one third of BD patients, with higher rates in male (44%) than in female (22%) patients (Di Florio et al., 2014). Overall, patients with addictions are 5–6 times more likely to have a history of BD compared to the general population (Kessler et al., 1997). Research has identified three subgroups of patients, presenting with AUD first, BD first, and both simultaneously. BD preceded by addiction may represent a milder illness form (Pacchiarotti et al., 2009).

Although the etiology of the BD-AUD comorbidity is poorly understood, several explanations have been put forward. Both BD and AUD are complex-trait conditions with overlapping etiopathophysiological pathways at the genetic, neurochemical, neurophysiologic and neuroanatomic levels (Farren et al., 2012). Shared genetic basis could confer risk for both BD and AUD (Johnson et al., 2009). Interestingly, this common genetic vulnerability would not be entirely driven by confounders, such as liability for anxiety disorders (Carmiol et al., 2014). Moreover, comorbid alcohol and substance use may also be a coping strategy by which patients try to manage (e.g., by self-treatment) their mood symptoms (Bizzarri et al., 2009; Do and Mezuk, 2013). BD and addictions may share common mechanisms, including high impulsivity, executive dysfunction, susceptibility to behavioral sensitization to stressors, as well as poor modulation of motivation and responses to rewarding stimuli (Swann, 2010; Tolliver and Hartwell, 2012). Indeed, high trait impulsivity may mediate some severe manifestations of this comorbidity (Swann et al., 2009; Nery et al., 2013).

At the clinical level, dual diagnosis seems to be mutually detrimental since addiction worsens the clinical presentation, course, prognosis and treatment of BD, and vice versa (Salloum and Thase, 2000). Compared to BD patients without addictions, dually diagnosed patients have earlier age of onset, poor treatment adherence and treatment response, longer and more frequent mood episodes and hospitalizations, more mixed episodes and rapid cycling, more comorbid anxiety disorders and greater impulsivity, and higher rates of aggressive behavior and suicide attempts (Swann, 2010; Tolliver and Hartwell, 2012; Nery et al., 2013). Comorbid addictions worsen functioning in BD, sometimes to that of SZ patients (Jaworski et al., 2011). Clearly, dual BD represents a prevalent, severe and difficult to treat subgroup of BD, but, surprisingly, little is known about its neurobiological and neurocognitive correlates (Nery et al., 2011).

## The neurocognitive dysfunction associated with alcohol use disorders

Chronic alcoholism exerts harmful effects on brain health and cognition, including significantly decreased cortical thickness (Momenan et al., 2012). In addition to brain atrophy, enlargement of the ventricles and sulci, as well as reductions in cerebral blood flow and glucose metabolism, particularly in prefrontal areas, have been described (Gupta and Warner, 2008). Moreover, chronic alcohol misuse has been consistently associated with widespread neurocognitive deficits, including episodic memory, attention, processing speed, visuospatial and motor abilities, verbal fluency, and executive functions, such as decision-making, problem-solving, working memory, and mental flexibility (Stavro et al., 2013; Bernardin et al., 2014). According to a recent meta-analysis, all these deficits were of moderate magnitude and IQ was the only domain not significantly affected by chronic alcoholism (Stavro et al., 2013). As many as 50-80% of patients show neurocognitive impairment, although there exists marked inter-individual variability in the nature and the severity of deficits (Bates et al., 2002; Bernardin et al., 2014). For instance, treatment-resistant heavy drinkers have more severe executive dysfunctions (Wollenweber et al., 2014). In the most severe and chronic cases, the clinical presentation may be dominated by cognitive features, such as confabulation, amnesia and confusional states (e.g., Wernicke-Korsakoff syndrome), as well as global cognitive deterioration (e.g., alcohol-related dementia).

Prospective studies suggest that abstinence from alcohol results in partial neurocognitive recovery, especially regarding sustained attention (Schulte et al., 2014). Overall, a widespread pattern of impairment seems to remain stable during the first year of sobriety and neurocognitive performance tends to normalize only after 1 year of abstinence (Stavro et al., 2013). However, certain functions, such as visuospatial abilities, may remain persistenly impaired even after longer periods of abstinence (Bernardin et al., 2014). Therefore, several memory rehabilitation strategies have been developed, although the field is still in its infancy (Svanberg and Evans, 2013).

Here we aim to review the literature that has examined the relative contribution of AUDs to the neurocognitive functioning of BD patients. Since both BD and AUDs have been associated with neurocognitive impairment on their own, patients with the BD-AUD comorbidity (e.g., dual diagnosis) may have more severe neurocognitive deficits than those with a single diagnosis.

To explore this hypothesis more thoroughly, we carried out a systematic literature review. Electronic databases (PubMed, Scopus, EMBASE) were searched through January 2015 using combinations of the following search terms: *bipolar disorder* cross-referenced with cognition, neurocogniti^*^ or neuropsycholog^*^ cross-referenced with *alcohol use disorder, alcohol abuse* or *alcohol dependence*. These searches retrieved 23, 63, and 389 hits, respectively. In addition, the reference lists of relevant papers were manually checked for further articles not previously identified. Studies comparing neuropsychological performance of BD subjects with/without AUDs were selected.

## The relative contribution of comorbid alcohol use disorders

So far, only eight studies met the selection criteria and have compared the neurocognitive functioning of BD patients with and without comorbid AUDs (van Gorp et al., 1998; Levy et al., 2008, 2012; Sanchez-Moreno et al., 2009b; van der Werf-Eldering et al., 2010; Shan et al., 2011; Chang et al., 2012; Marshall et al., 2012). The major characteristics of these studies are shown in Table 1.

**Table 1:** **Main characteristics of studies included in the review**.

**Study**	**Country**	**Sample description**		**Measures of alcohol consumption**	**Urine screening test to exclude other SUDs**	**Specific comments**
		**Type of BD**	**Phase of BD**			
van Gorp et al., 1998	USA	25 BD (type not specified) -12 with past AD - 13 without AD 22 HC	All patients euthymic (HRSD<7 and YMRS<6 for 3 consecutive monthly assessments)	None	Yes	Dual patients should be abstinent at least 6 months (mean duration > 8 years) All subjects were male
Levy et al., 2008	USA	63 BD (all type I) - 13 with current AD (past 6 months) - 9 with remitted AD - 41 without history of SUD No HC	All inpatients with acute mood episodes BDI <15, BHS <10 and YMRS <15	Quantity and frequency (e.g., number of standard alcoholic drinks consumed in the past month, days alcohol was used in the past month)	No	Detoxification upon admission was not required for patients with current AD History of SUD other than alcohol was allowed for the dual groups Substance use measures (ASI, AUDIT)
Sanchez-Moreno et al., 2009b	Spain	65 BD (51 type I) - 30 with history of AB/AD - 35 without AB/AD 35 HC	All euthymic outpatients with 6 consecutive months of remission (HRSD ≤ 8 and YMRS ≤ 6)	None	Yes	Dual patients should be abstinent for at least 1 year History of SUD other than alcohol was excluded as per DSM
van der Werf-Eldering et al., 2010	Netherlands	110 BD (91 type I, 19 type II) - 21 with lifetime AUD (13 with current AUD) 75 HC	Outpatients either euthymic (*n* = 46) or with mild or moderate depressive symptoms (*n* = 64) Severe depressive symptoms (IDS>38) and manic symptoms (YMRS>7) were exclusion criteria	None	No	Patients with severe AUD (currently needing treatment in specialized setting) were excluded
Shan et al., 2011	Taiwan	69 BD (all type II) - 19 BD with history of AB/AD - 28 BD without AB/AD 22 HC	All patients in remission for at least 2 weeks (HRSD < 7 and YMRS < 6)	For dual patients: g/day	No	History of SUD other than alcohol was excluded as per DSM Duration of abstinence was not reported
Chang et al., 2012	Taiwan	38 BD-I: - 16 with comorbid AD - 22 without history of AD 56 BD-II - 18 with comorbid AD - 38 without history of AD 29 HC	Same as Shan et al., 2011	None	No	Same as Shan et al., 2011 All subjects were male
Levy et al., 2012	USA	55 BD (all type I) - 21 with comorbid AD (previous year) - 34 without SUD No HC	At baseline, all inpatients with acute mood episodes (34 mania, 12 mixed, 9 depression)	Same as Levy et al., 2008	Yes (only at 3-month follow-up)	Detoxification upon admission was not required for patients with AD History of SUD other than alcohol was allowed for the dual group
Marshall et al., 2012	USA	256 BD (201 type I, 36 type II, 19 NOS) - 158 with history of SUD (130 with alcohol) - 98 without SUD 97 HC	Outpatients and inpatients without manic symptoms (YMRS ≤ 7)	None	No	Almost half of the SUD group met criteria for multiple substances Subjects with active or current substance dependence were excluded

*AB, alcohol abuse; AD, alcohol dependence; ASI, Addiction Severity Index; AUD, Alcohol Use Disorder; AUDIT, Alcohol Use Disorder Identification Test; BD, bipolar disorder; BDI, Beck Depression Inventory; BHS, Beck Hopelessness Scale; DSM-IV, Diagnostic and Statistical Manual—4th edition; HC, healthy control; HRSD, Hamilton Rating Scale for Depression; IDS, Inventory of Depressive Symptomatology; NOS, not otherwise specified; SUD, substance use disorder; YMRS, Young Mania Rating Scale*.

In a pioneer work, van Gorp et al. (1998) examined 12 BD patients with past history of alcohol dependence, 13 BD patients without such comorbidity, and 22 healthy controls. Only males were recruited and all outpatients were euthymic at the time of neurocognitive assessment. Both BD groups showed verbal memory deficits, whereas only the dual group had an additional executive deficit measured by the number of completed categories in the Wisconsin Card Sorting Test (WCST). Moreover, neurocognitive functioning was negatively correlated with lifetime duration of manic or depressive episodes, suggesting that patients with greater illness burden had poorer performances.

Interestingly, it took one decade for the field to revisit this topic. Levy et al. (2008) compared three groups of BD-I inpatients, who were admitted mostly due to manic episodes. A first group with current alcohol dependence (*n* = 13), a second group in full remission (e.g., during at least 1 year) from alcohol dependence (*n* = 9), and a third non-dual group without history of SUDs (*n* = 41). Those with current alcohol dependence were significantly more impaired than the non-dual group in measures of visual memory and verbal memory. Moreover, both dual BD groups performed significantly worse than non-dual BD patients on executive functions measured by the Stroop test and WCST. These findings would suggest that the BD-AUD comorbidity is associated with more severe mnemonic and executive dysfunction, and that the neurocognitive consequences of past AUDs may persist despite sustained abstinence from alcohol. However, the presence of subacute, residual mood symptoms during examination before hospital discharge may increase the severity of deficits found in this study.

Another study by the same research group focused on cognition during the course of early remission from a severe mood episode (Levy et al., 2012). This 3-month, follow-up study compared 21 BD patients with AUDs in the previous year and 34 BD patients without a history of SUDs. Dually diagnosed patients performed worse on measures of verbal memory, visual memory, and executive functioning on both assessments and showed a poorer neurocognitive recovery relative to those without SUDs. These findings underscore the special needs of BD-AUD patients in terms of intensive treatment and support aimed to achieve early recovery after relapses. To that end, detailed and serial neuropsychological evaluations during this critical period remain as a backbone.

Consistently, another study (Sanchez-Moreno et al., 2009b) found that euthymic BD patients with (*n* = 30) and without (*n* = 35) previous history of AUDs performed poorer than healthy controls (*n* = 35) in verbal memory and executive functions, regardless alcohol history. However, patients with previous alcohol misuse were more impaired in the Stroop interference task, suggesting greater difficulties in the inhibitory control of inadequate behaviors, which may be related to higher impulsivity and probably to higher risk of other addictive behaviors. Dual patients were requested to be abstinent for at least 1 year but time of abstinence was not recorded. BD-II patients were also recruited, and this is particularly relevant since type II is also significantly associated with neurocognitive impairments and AUDs (McElroy et al., 2001; Solé et al., 2012).

In this regard, a Taiwanese study focused only on type-II BD (Shan et al., 2011). The authors compared 19 patients with comorbid AUD, 28 patients without comorbid AUD, and 22 healthy controls. All participants were alcohol-free at least 24 h before examination and BD patients were euthymic. Compared to the other two groups, dual patients performed significantly worse on tasks of visual memory, verbal memory, attention, psychomotor speed, and executive functioning. In addition, working memory was impaired in both BD groups, although more so in dual patients. However, the clinical groups were not balanced regarding gender, educational level and number of hospitalizations, so a potential influence of these relevant variables on neurocognitive results cannot be entirely ruled out.

A subsequent study from the same research group (Chang et al., 2012) explored whether the neurocognitive effects of comorbid AUD is similar or different depending on the type of BD. To this end, BD-AUD patients (type I = 16; type II = 18) were compared with non-dual BD patients (type I = 22; type II = 38). The four clinical groups showed widespread neurocognitive deficits compared to healthy controls (*n* = 29) even during euthymia. Dually diagnosed patients performed significantly worse than non-dual BD patients. Of note, non-dual BD-I patients showed widespread deficits, especially in tests of attention/concentration and working memory and, whereas non-dual BD-II patients performed similarly to controls. The authors concluded that alcohol misuse seems to exert greater neurocognitive impact on BD-I. However, only male patients were recruited in this study and subjects from Asia have specific features related to alcohol consumption.

In the study with the largest sample (*n* = 353) so far, Marshall et al. (2012) evaluated 98 non-dual BD patients, 158 BD patients with comorbid addictions (130 of whom had AUDs) and 97 healthy subjects. Compared to controls, BD patients had a widespread dysfunction in areas of motor speed and dexterity, visual memory, processing speed and verbal fluency. Moreover, the dual group performed significantly worse than the non-dual group on tasks of visual memory and reasoning.

On the contrary, only one study has concluded that alcoholism was not associated with neurocognition among 185 BD patients (van der Werf-Eldering et al., 2010). However, this result was based on a *post-hoc* analysis. Moreover, the authors did not aim to compare dual and non-dual patients and even the rate of comorbid AUDs was not reported.

## Implications for clinical practice and research

Taken together, most studies have found that BD patients with current or past history of comorbid AUDs show more severe and/or widespread neurocognitive deficits than their non-dual counterparts. Although there is marked variability in the findings, this impairment mostly involves the broad domains of verbal memory and executive cognition. Moreover, the reviewed literature further confirms that BD itself (e.g., non-dual BD) is associated with a significant neurocognitive dysfunction, regardless mood state (Kurtz and Gerraty, 2009; Bourne et al., 2013). Cognitive dysfunction would be another phenotypic dimension common to BD and AUD. Collectively, these findings imply either that alcohol misuse poses an additional neurocognitive tax to that intrinsic to BD itself or that the BD-AUD comorbidity is a more severe form of illness associated with greater cognitive dysfunction.

The conclusion of this systematic review must be regarded as tentative given the reduced number and heterogeneity of extant studies. The former may result from neuropsychological studies usually excluding patients based on concurrent or recent misuse of alcohol and other substances. Several methodological aspects of the original studies must be also limit the generalization of present findings. Firstly, the sample size in most cases is relatively modest, especially regarding the comorbid BD-AUD groups, which may reflect the difficulty in recruiting clinically stable and motivated patients who consent going through burdensome evaluations. Secondly, the timing of examination widely differs between studies and not all of them have assessed patients during euthymia. Doing so is currently considered a gold standard in neurocognitive research of BD (Bourne et al., 2013), but the distinct features of dual patients likely advices a less stringent approach. In this regard, proximity to an acute episode, as well as use of higher doses and combinations of pharmacological agents during admissions have been associated with worse neurocognitive performances (Balanzá-Martínez et al., 2010). Thirdly, key clinical variables, such as number of past episodes and, more importantly, time of abstinence were not recorded in all studies. Fourthly, the recruitment of homogenous samples according to gender or race may introduce another bias. Methodological refinement and standardization of procedures would allow gaining a deeper understanding of this phenomenon. Fifthly, concomitant addiction to other substances, such as cannabis, may also contribute to neurocognitive dysfunctions in BD (Cahill et al., 2006) and are beyond the scope of the present review. Similarly, complex patterns may also result from interactions with medical comorbidities and deserve further study. Cardiovascular and metabolic conditions, such as hypertension or type 2 diabetes, which are usually comorbid with both BD and AUD, are well known risk factors for cognitive deterioration (Durazzo et al., 2008). Lastly, except a 3-month follow-up study (Levy et al., 2012), most research so far has been cross-sectional. Therefore, longitudinal designs will aid to better establish the temporal relationship between neurocognitive status and the clinical features of BD and AUD.

At the clinical level, the present findings have several implications for diagnosis, treatment and prognosis. Comorbid addictive disorders, including AUDs, are a potentially treatable risk factor. Early detection and intervention is a pressing need in BD (Conus et al., 2014), and this clearly turns mandatory for dual BD, especially among young people (Hermens et al., 2013). The ultimate goal of treatments is to improve patients' functional outcomes and quality of life. This seems achievable since remission from both alcohol dependence and BD has been associated with significant improvements in quality of life compared to non-remission (Rubio et al., 2013). Moreover, absence of AUD was associated with better neurocognitive recovery during the early course of BD (Torres et al., 2014). However, few pharmacological and behavioral interventions have effectively addressed the clinical management of dual populations, probably because they may not be well-suited for this cognitively-impaired population (Bradizza et al., 2014). Indeed, neurocognitive dysfunction may represent a barrier for dual patients to benefit from psychosocial treatments, and probably also from pharmacological agents through indirect effects on diminished adherence (Martinez-Aran et al., 2009; Vieta et al., 2012; Jónsdóttir et al., 2013; Fagan et al., 2015). Preventative and treatment strategies should target neurocognitive dysfunction as a major driver of patients' functional outcomes (Tabarés-Seisdedos et al., 2008).

These findings also suggest that future neurocognitive studies of BD should take into account the potential confounding effects of comorbid AUDs, including past exposures to psychoactive substances (Savitz et al., 2005). In our opinion, two additional implications for research merit further discussion. Cosci and Fava (2011) have recently proposed an alternative strategy to examine dual diagnosis based on clinimetric methods, helped by staging and evaluation of subclinical symptoms. According to these authors, clinical staging may provide a more holistic approach to dual BD patient's problematic areas, including neurocognitive dysfunctions. Here we suggest that BD-AUD may similarly benefit from the application of another holistic perspective—systems biology.

Several staging models have been put forward to explain the progressive deterioration that takes place in a significant proportion of BD patients (Kapczinski et al., 2014). Comorbid conditions, including addictions, are predicted to be associated with greater illness progression, chronicity and deterioration (Kapczinski et al., 2009). Kindling, sensitization and allostatic load may explain the progressive course and negative outcomes of dual BD (Post and Kalivas, 2013; Pettorruso et al., 2014). Early life (e.g., childhood) adversity and stressors play a major role in the onset and relapses of both BD and AUD, and also explain the high comorbidity between them (Post and Leverich, 2006; Post and Kalivas, 2013).

However, no study has examined the neurocognitive burden of comorbid AUDs according to clinical staging (e.g., comparing early- vs. late-stage BD patients). On the other hand, few staging models for addictions exist (Langenbucher and Chung, 1995; Favrat et al., 2002) and none has been developed specifically for dual diagnosis (Cosci and Fava, 2011). In all, we propose the application of staging to better understand the neurocognitive dysfunction associated with either BD or AUD alone, and their comorbid presentation.

A systems biology approach, integrating –omics data with bioinformatical tools, aims to gain deeper insights into the etiopathophysiology of a certain disease, which in turn may provide new therapeutic targets that should be translated into clinical practice (Hoertel et al., 2013). The potential relevance of systems medicine for AUD (Spanagel et al., 2013; Gorini et al., 2014) and BD (Frangou, 2014; McIntyre et al., 2014) has been recently proposed. We agree with McIntyre et al. (2014) that this approach may be particularly relevant for BD with comorbid conditions. Specifically, systems biology provides an exciting opportunity to better understand the BD-AUD comorbidity at different levels. Unraveling the genes and proteins involved in the vulnerability to BD-AUD is relevant to inform on the subserving molecular and cellular mechanisms and to identify novel treatments and molecules for the management of this comorbidity. This is clearly relevant since many dual BD patients may receive suboptimal treatments. This approach may also prove fruitful to refine current nosology of dual diagnosis based on more biologically informed grounds (Frangou, 2014). In sum, the bipolar-addiction comorbidity may benefit from the application of holistic approaches, such as staging and systems biology.

## Author contributions

All the authors have been sufficiently involved in the submitted study and have approved the final paper.

### Conflict of interest statement

Dr. Balanzá-Martínez has received grants and served as consultant, advisor or CME speaker during the last four years for the following entities: Almirall; Angelini; AstraZeneca; Bristol-Myers-Squibb; Janssen; Juste; Lundbeck; Otsuka; Spanish Ministry of Science and Innovation (CIBERSAM); and Fundación Alicia Koplowitz. Dr. Crespo-Facorro declares that he has no conflict with the contents of this paper. Dr. González-Pinto has received grants from or served as consultant, adviser, or CME speaker for Almirall, AstraZeneca, Bristol-Myers Squibb, Cephalon, Eli Lilly, GlaxoSmithKline, Janssen-Cilag, Lundbeck, Merck, Novartis, Otsuka, Pfizer, Sanofi- Aventis, Schering-Plough, and the Spanish Ministry of Science and Innovation (CIBERSAM). Dr. Vieta has received grants, CME-related honoraria, or consulting fees from Alexza, Almirall, AstraZeneca, Bristol-Myers Squibb, Cephalon, Eli Lilly, Ferrer, Forest Research Institute, Gedeon Richter, GlaxoSmith-Kline, Janssen, Janssen-Cilag, Jazz, Johnson and Johnson, Lundbeck, Merck, Novartis, Organon, Otsuka, Pfizer, Pierre-Fabre, Qualigen, Roche, Sanofi-Aventis, Schering-Plough, Servier, Shire, Solvay, Takeda, Teva, CIBERSAM, the Seventh European Framework Programme (ENBREC), the Stanley Medical Research Institute, United Biosource Corporation, and Wyeth.
